# Hemoglobin is associated with hypertension-mediated cardiovascular damages in hypertensive patients with high-altitude polycythemia

**DOI:** 10.1007/s11739-024-03800-7

**Published:** 2024-11-07

**Authors:** Xiangyu Yang, Hongwei Li, Jie Zhang, Xiajiao Yang, Qianqiu Che, Zhengyao Cai, Yuting Cao, Yongxing Fu, Jinghua Zhao, Xin Zhang, Xiaoping Chen, Liming Zhao

**Affiliations:** 1 Department of Cardiology, West China Hospital, Sichuan University, Chengdu, 610041 China; 2 Department of Cardiology, Hospital of Chengdu Office of People’s Government of Tibetan Autonomous Region, Chengdu, 610041 China

**Keywords:** High-altitude polycythemia, Hypertension, Hypertension-mediated organ damage, Echocardiography, Flow-mediated dilation

## Abstract

High-altitude polycythemia (HAPC) is a pathological state resulting from maladaptation to prolonged high-altitude exposure, posing significant risks to the cardiovascular health of highlanders. However, its influence on hypertension-mediated organ damages (HMODs) in hypertensive individuals remains unclear. We recruited hypertensive patients residing at altitudes above 2500 m for over 3 years. A case–control matching was conducted in a 1:1 ratio between hypertensive patients with and without HAPC, based on gender and age. Echocardiography, carotid artery ultrasound, and brachial flow-mediated dilation (FMD) were measured as HMODs. A total of 88 hypertensive patients were included in the analysis, with 44 with HAPC and 44 without HAPC. Patients with HAPC showed significantly higher hemoglobin (HGB) levels (217.82 ± 17.34 vs. 160.16 ± 13.25, *P*<0.001), a larger left atrium (LA) diameter (35.36 ± 4.25 vs. 33.09 ± 3.55, *P* = 0.008), and a higher proportion of impaired FMD (95.45% vs. 79.55%, *P* = 0.049) compared to those without HAPC. No significant differences were found between the two groups in diastolic function parameters, left ventricular mass index (LVMI), relative wall thickness (RWT), or intima-media thickness (IMT). After adjusting for age, gender, and other confounding factors, HGB remained significantly associated with LA diameter (*β* = 0.034, *P* = 0.023) and impaired FMD (OR = 1.034, 95% CI 1.001–1.069). After matching for age and gender, hypertensive patients with HAPC exhibited a significantly larger LA diameter and a higher prevalence of impaired FMD compared to those without HAPC. Additionally, HGB was identified as an independent risk factor for both increased LA diameter and impaired FMD in hypertensive patients with HAPC.

## Introduction

Due to the unique hypobaric hypoxic environment at high altitudes, individuals residing in such regions face a greater challenge to cardiovascular health [1]. Chronic exposure to high-altitude environments triggers a series of physiological adaptations aimed at mitigating the effects of low oxygen availability, primarily through hematological changes [2]. However, when these adaptive mechanisms exceed the body’s compensatory capacity, maladaptation occurs, resulting in chronic mountain sickness (CMS). Epidemiological data indicate that the prevalence of CMS ranges from 5 to 33% worldwide. High-altitude polycythemia (HAPC), one of the most important aspects of CMS, is defined by hemoglobin concentrations exceeding 190 mg/dL in females and 210 mg/dL in males [3].

Arterial hypertension represents a significant global health challenge, contributing substantially to the public health burden. Previous epidemiological surveys have consistently shown a higher prevalence of hypertension in high-altitude regions compared to lowland areas [4]. Prolonged elevation of blood pressure (BP) can lead to a spectrum of hypertension-mediated organ damages (HMODs), which is an important risk factor for the development of cardiovascular diseases and the occurrence of cardiovascular events [5]. Consequently, managing BP and assessing HMODs in hypertensive patients residing in high-altitude regions are essential strategies for preventing cardiovascular diseases and reducing associated mortality.

In individuals with HAPC, the cardiovascular system plays a significant role in the body’s integrated response to hypoxia [6]. Previous studies have demonstrated that individuals living at high altitudes with HAPC face a heightened cardiovascular risk compared to healthy ones [7]. However, there is limited data regarding hypertensive patients with coexisting HAPC. It remains unclear whether HAPC affects BP control in patients with hypertension or exacerbates HMODs in these individuals. Therefore, in this study, we employed a case–control design to investigate the extent of HMODs in hypertensive patients with and without concurrent HAPC, and to explore potential risk factors associated with the exacerbation of HMODs in these patients.

## Methods

### Study population

The study was conducted between 2019 and 2023 at both the outpatient and inpatient departments of the Hospital of Chengdu Office of People’s Government of Tibetan Autonomous Region and West China Hospital, Sichuan University. Inclusion criteria were as follows: (1) Residents living at altitudes above 2500 m for more than 3 years; (2) Patients diagnosed with hypertension with BP < 180/110 mmHg without antihypertensive medication treatment; (3) Age between 30 and 55 years old. Exclusion criteria included: (1) Patients with hematological diseases such as anemia, polycythemia vera, or myeloproliferative neoplasms; (2) Patients with secondary hypertension; (3) Patients with confirmed atherosclerotic cardiovascular diseases (ASCVDs), atrial fibrillation, congenital heart disease, or heart failure (ejection fraction < 50%); (4) Patients with liver or kidney dysfunction; (5) Patients with diabetes mellitus.

**Fig. 1:**
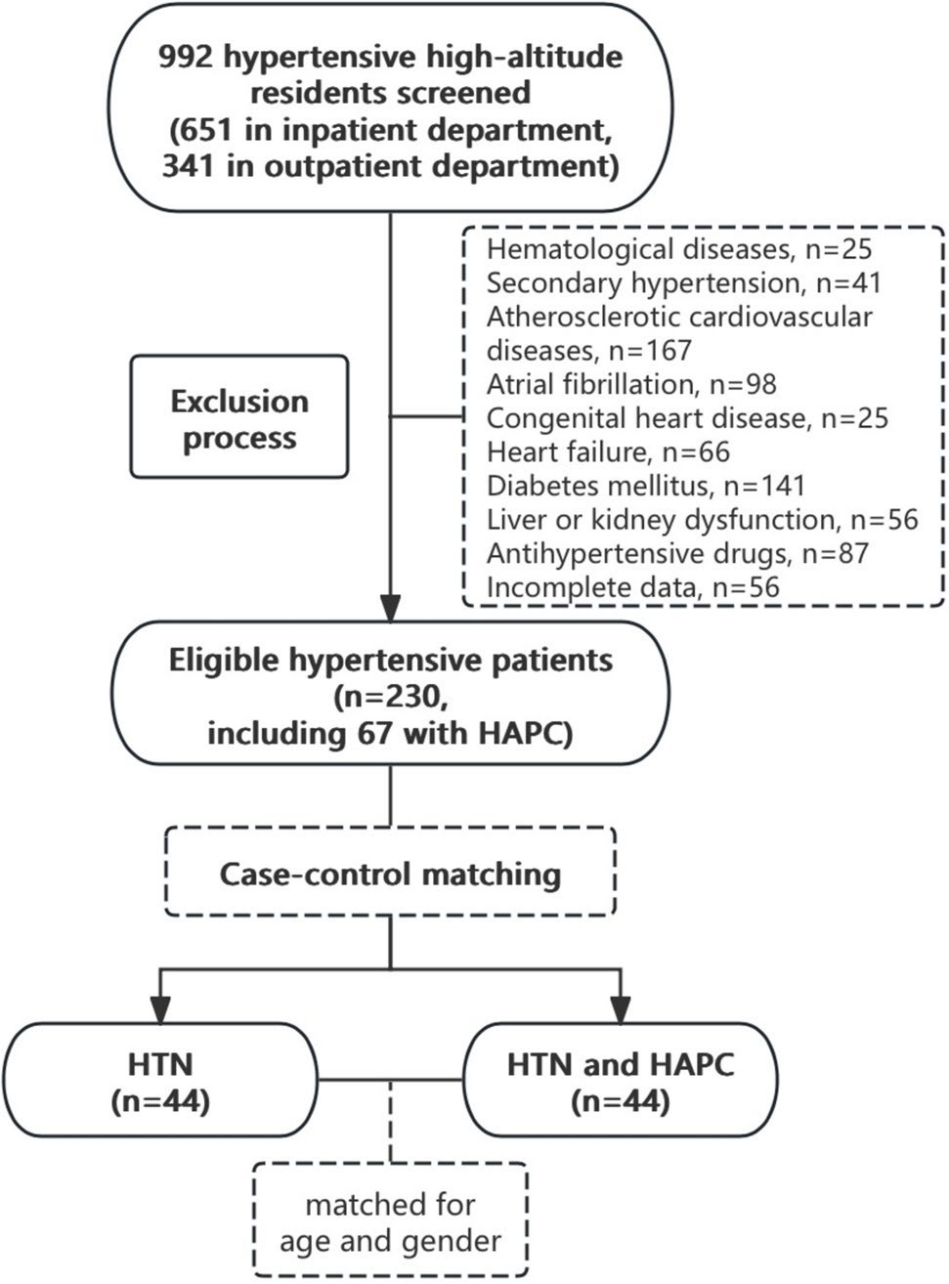
Flowchart

A total of 230 patients with hypertension were enrolled in this study, 67 of whom were diagnosed with both hypertension and HAPC. Case–control matching was performed in a 1:1 ratio between hypertensive patients with and without HAPC according to their gender and age. Eventually, 44 hypertensive patients with HAPC and 44 without HAPC were successfully matched and included in the final analysis (Fig. 1). Informed consent was obtained from all participants, and the study was approved by the Ethics Committee of West China Hospital, Sichuan University.

Definition of HAPC: Hemoglobin ≥ 210 g/L for males, and ≥ 190 g/L for females [3].

### Data collection

All patients were required to complete a questionnaire upon enrollment, which included basic demographic information such as gender, age, smoking and drinking status, and medical history, etc. Physical examinations were performed by trained nurses. Height and weight measurements were precise to 0.1 cm and 0.1 kg, respectively. Body mass index (BMI) was calculated as BMI = Weight/Height^2^ (kg/m^2^). Obesity was defined as BMI ≥ 28 kg/m^2^ [8].

Office blood pressure (OBP) was measured after a 5-min rest for each patient. BP measurements were conducted using a validated and calibrated upper-arm electronic sphygmomanometer (Omron HBP-1100). Three consecutive measurements were recorded, with a 1-min interval between each. The average of the last two BP measurements was used for statistical analysis [9].

Blood samples were collected from patients following an 8-h fasting. Laboratory parameters included hemoglobin (HGB), fasting blood glucose (FBG), creatinine (CREA), uric acid (UA), total cholesterol (TC), triglycerides (TG), high-density lipoprotein cholesterol (HDL-C), and low-density lipoprotein cholesterol (LDL-C). All laboratory assessments were performed at Hospital of Chengdu Office of People’s Government of Tibetan Autonomous Region. Estimated glomerular filtration rate (eGFR) was calculated according to the 2021 CKD-EPI eGFRcr equation [10]. Hyperuricemia was defined as serum UA ≥ 420 μmol/L for males, and serum UA ≥ 360 μmol/L for females [11].

### Echocardiography measurement

Echocardiography was performed by professional cardiac ultrasound technicians at the Hospital of Chengdu Office of People’s Government of Tibetan Autonomous Region. All the procedures were conducted according to the guidelines from the American Society of Echocardiography [12]. Relative wall thickness was calculated as: (2 × posterior wall thickness)/(left ventricular internal diameter at end-diastole). RWT > 0.42 represents for concentric remodeling [13]. Left ventricular mass (LVM), body surface area (BSA), and left ventricular mass index (LVMI) were calculated according to the following formulae.

$${\text{LVM}} = 0.8 \times 1.04 \times [({\text{IVST}} + {\text{LVPWT}} + {\text{LVEDD}})^{3} - {\text{LVEDD}}^{3} ] + 0.6.$$

$${\text{BSA}} = 0.0061 \times {\text{height}}({\text{cm}}) + 0.0125 \times {\text{weight}}({\text{kg}}) - 0.1529.$$

$${\text{LVMI}} = {\text{LVM}}/{\text{BSA}}.$$

### Carotid artery ultrasound

Carotid intima-media thickness (IMT) was measured by professional technicians at the Hospital of Chengdu Office of People’s Government of Tibetan Autonomous Region, using a Philips CX-50 color Doppler ultrasound system, equipped with an L12-3 probe and Qlab software. IMT was assessed as the distance between the intimal and adventitial layers of the carotid artery at three locations: the bifurcation, 1–1.5 cm proximal to the bifurcation, and 1–1.5 cm distal to the bifurcation during end-diastole. Areas with atherosclerotic plaques were carefully avoided. The average value of the measurements from these three locations was calculated as the left or right IMT dimension [12].

### Brachial flow-mediated dilation measurement

Brachial artery flow-mediated dilation (FMD) was measured by professional physicians in accordance with current guidelines [14]. The measurements were conducted using a specialized ultrasound machine equipped with an automated tracking system (UNEXEF18VG, UNEX Co., Nagoya, Japan) after a supine rest of 10–15 min in a quiet, temperature-controlled room for all patients. Firstly, the cuff was placed on the forearm of each patient. The baseline diameter (D1) of the brachial artery, located 5–10 cm above the elbow, was recorded for at least 30 s. Then the cuff was inflated to approximately 200 mmHg to occlude the artery for 5 min. Following cuff deflation, the post-deflation diameter (D2) of the artery was continuously measured for over 3 min. FMD was calculated as (D2 − D1)/D1 × 100% using the device’s integrated algorithms.

Impaired FMD was defined as FMD < 6.5% [15].

### Statistical analysis

Metric data following a normal distribution were presented as mean ± SD. Those deviated from a normal distribution were presented as median and interquartile range. Categorical data were reported as frequencies and percentages (*n*, %). Comparisons of normally distributed metric data were conducted using the *t* test, whereas non-normally distributed data were analyzed using the non-parametric Kruskal–Wallis test. Rates and proportions were compared using the Chi-square test. Univariate and multivariate linear regression analyses were used to assess the association between hemoglobin levels and left atrium (LA) diameter. Univariate and multivariate logistic regression analyses were used to identify risk factors for impaired FMD in the participants. The results were presented as odds ratio (OR) with 95% confidential intervals (95% CIs). Statistical analysis was performed using SPSS version 27.0 (IBM Corp., Armonk, New York, USA). *P* value <0.05 was considered statistically significant.

## Results

### Baseline characteristics of the participants

A total of 992 hypertensive residents living at high altitudes were screened. After applying the inclusion and exclusion criteria, 230 patients met the eligibility criteria, including 67 patients with hypertension and concomitant HAPC. Following case–control matching in a 1:1 ratio based on age and gender, 88 hypertensive patients were ultimately included in the study, with 44 in the HAPC group and 44 in the hypertension combined with the HAPC group (Fig. 1). Compared to patients without HAPC, those with HAPC exhibited significantly higher HGB levels (217.82 ± 17.34 vs. 160.16 ± 13.25, *P* < 0.001). However, mean office systolic blood pressure (SBP) (129.41 ± 15.00 vs. 137.66 ± 18.89, *P* = 0.026), and the proportion of drinkers (38.64% vs. 65.91%, *P* = 0.018) were significantly lower in patients with HAPC than those without HAPC. No significant differences were observed in eGFR, hospitalization rates, or other parameters between the two groups (all *P* values ≥0.05, Table 1).

**Table 1:** Baseline characteristics of the participants

Characteristics	Overall (*n* = 88)	HTN (*n* = 44)	HTN and HAPC (*n* = 44)	*P* value
Male, *n* (%)	80 (90.91)	40 (90.91)	40 (90.91)	1.000
Age (years)	47.99 ± 5.60	48.09 ± 5.80	47.89 ± 5.48	0.865
BMI (kg/m^2^)	27.43 ± 4.07	26.92 ± 3.10	27.93 ± 4.84	0.248
Smoking, *n* (%)	34 (38.64)	14 (31.82)	20 (45.45)	0.274
Drinking, *n* (%)	46 (52.27)	29 (65.91)	17 (38.64)	0.018*
HGB (g/L)	188.99 ± 32.80	160.16 ± 13.25	217.82 ± 17.34	<0.001^#^
FBG (mmol/L)	5.31 ± 0.75	5.28 ± 0.65	5.35 ± 0.86	0.689
CREA (mmol/L)	72.00 ± 17.79	74.17 ± 17.98	69.82 ± 17.53	0.253
UA (mmol/L)	451.77 ± 95.74	442.11 ± 87.78	461.43 ± 103.21	0.347
TG (mmol/L)	1.81 ± 0.98	1.88 ± 0.97	1.74 ± 0.99	0.496
TC (mmol/L)	4.68 ± 1.27	4.79 ± 1.57	4.57 ± 0.86	0.430
HDL-C (mmol/L)	1.10 ± 0.24	1.14 ± 0.24	1.06 ± 0.23	0.127
LDL-C (mmol/L)	2.71 ± 0.83	2.62 ± 0.57	2.73 ± 0.70	0.385
eGFR (ml/min/1.73 m^2^)	105.16 ± 14.82	103.43 ± 12.85	106.88 ± 16.53	0.277
SBP (mmHg)	133.53 ± 17.46	137.66 ± 18.89	129.41 ± 15.00	0.026*
DBP (mmHg)	91.08 ± 12.42	92.34 ± 13.26	89.82 ± 11.53	0.344
Obesity, *n* (%)	31 (35.23%)	14 (31.82%)	17 (38.64%)	0.328
Hyperuricemia, *n* (%)	57 (64.77%)	28 (63.64%)	29 (65.91%)	0.500
Hospitalization, *n* (%)	55 (62.50%)	26 (59.09%)	29 (65.91%)	0.330

Values are expressed as mean ± SD or number of participants (%)

*HTN* hypertension, *HAPC* high-altitude polycythemia, *BMI* body mass index, *HGB* hemoglobin, *FBG* fasting blood glucose, *CREA* creatinine, *URIC* uric acid, *TG* triglycerides, *TC* total cholesterol, *HDL-C* high-density lipoprotein-cholesterol, *LDL-C* low-density lipoprotein-cholesterol, *eGFR* estimated glomerular filtration rate, *SBP* systolic blood pressure, *DBP* diastolic blood pressure

* represents *P* < 0.05

^#^ represents *P* < 0.01

### Hypertension-mediated target organ damages of the participants

As for hypertension-mediated target organ damages, we found a significantly larger LA diameter (35.36 ± 4.25 vs. 33.09 ± 3.55, *P* = 0.008), a higher proportion of impaired FMD (95.45% vs. 79.55%, *P* = 0.049), and a lower mean FMD (4.73 ± 1.22 vs. 5.25 ± 1.18, *P* = 0.047) in hypertensive patients with HAPC compared to those without HAPC. However, no significant differences were observed between the two groups regarding *E*/*A*, *E*/*e*′, RWT, LVMI, IMT, or other parameters (all *P* values ≥0.05, Table 2).

**Table 2:** Hypertension-mediated target organ damages of the participants

Characteristics	Overall (*n* = 88)	HTN (*n* = 44)	HTN and HAPC (*n* = 44)	*P* value
IMT-L (mm)	0.68 ± 0.17	0.69 ± 0.15	0.67 ± 0.19	0.612
IMT-R (mm)	0.78 ± 0.21	0.70 ± 0.15	0.86 ± 0.11	0.358
Carotid plaque, *n* (%)	38 (43.18%)	20 (45.45%)	18 (40.91%)	0.830
LA (mm)	34.23 ± 4.05	33.09 ± 3.55	35.36 ± 4.25	0.008^#^
LV (mm)	47.06 ± 3.83	46.45 ± 3.11	47.66 ± 4.38	0.141
IVS (mm)	9.31 ± 1.53	9.16 ± 1.58	9.45 ± 1.49	0.369
LVPW (mm)	8.91 ± 1.07	8.95 ± 1.16	8.86 ± 1.00	0.695
AAO (mm)	32.50 ± 3.41	32.75 ± 3.79	32.25 ± 3.01	0.495
*E*/*A*	1.05 ± 0.35	1.06 ± 0.30	1.04 ± 0.39	0.820
*E*/*e*′	12.26 ± 3.98	12.67 ± 3.76	11.86 ± 4.19	0.340
RWT	0.38 ± 0.05	0.39 ± 0.04	0.38 ± 0.06	0.398
LVMI (g/m^2^)	84.94 ± 16.84	76.48 ± 21.14	79.47 ± 16.41	0.460
FMD (%)	4.99 ± 1.22	5.25 ± 1.18	4.73 ± 1.22	0.047*
Concentric remodeling, *n* (%)	14 (15.91%)	8 (18.18%)	6 (13.64%)	0.772
LVH, *n* (%)	9 (10.23%)	5 (11.36%)	4 (9.10%)	1.000
Impaired FMD, *n* (%)	77 (87.50)	35 (79.55)	42 (95.45)	0.049*

Values are expressed as mean ± SD or number of participants (%)

*IMT-L* intima-media thickness (left), *IMT-R* intima-media thickness (right), *LA* left atrium, *LV* left ventricle, *IVS* interventricular septal wall, *LVPW* left ventricular posterior wall, *AAO* ascending aorta, *E* early diastolic inflow velocity, *A* atrial systolic inflow velocity, *e*′ early diastolic mitral annular velocity, *RWT* relative wall thickness, *LVMI* left ventricular mass index, *LVH* left ventricular hypertrophy, *FMD* flow-mediated dilatation

* represents *P* < 0.05

^#^ represents *P* < 0.01

### Association of HGB with LA diameter in the participants

**Fig. 2:**
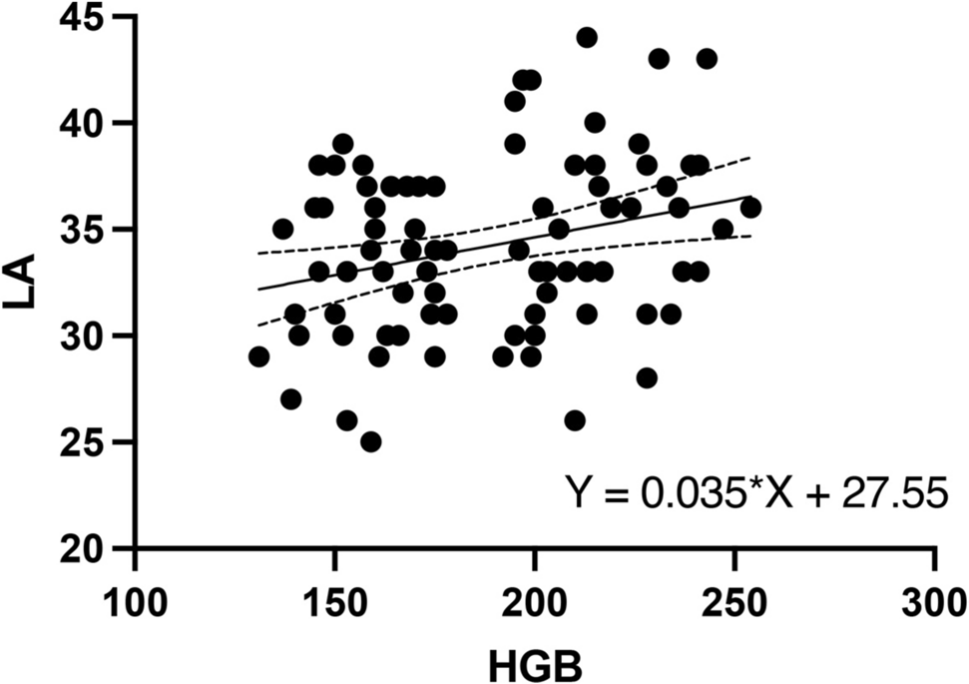
Univariate linear regression analysis of HGB and LA diameter

In the univariate analysis, HGB was significantly correlated with LA diameter (*β* = 0.035, *P* = 0.007, Fig. 2). After performing collinearity diagnostics and adjusting for confounding factors including age, gender, smoking and drinking status, BMI, FBG, CREA, URIC, LDL-C, and SBP in the multivariate analysis, HGB remained significantly associated with LA diameter (*β* = 0.034, *P* = 0.023).

### Clinical characteristics of the participants with or without impaired FMD

According to FMD < 6.5%, all participants were divided into the normal FMD (*n* = 11) and the impaired FMD group (*n* = 77). Compared to the patients with normal FMD, those with impaired FMD showed significantly higher HGB levels (192.22 ± 32.35 vs. 166.36 ± 27.67, *P* = 0.014), and lower FMD values (4.68 ± 0.91 vs. 7.17 ± 0.81, *P* < 0.001). Nevertheless, no significant differences were observed in gender, age, eGFR, or other parameters between the two groups (all *P* values >0.05, Table 3).

**Table 3:** Characteristics of the participants by impaired FMD

Characteristics	Normal FMD (*n* = 11)	Impaired FMD (*n* = 77)	*P* value
Male, *n* (%)	9 (81.82)	71 (92.21)	0.261
Age (years)	47.73 ± 6.00	48.03 ± 5.59	0.870
BMI (kg/m^2^)	27.09 ± 3.36	27.48 ± 4.28	0.769
Smoking, *n* (%)	3 (27.27)	16 (40.26)	0.519
Alcohol, *n* (%)	5 (45.45)	41 (53.25)	0.751
HGB (g/L)	166.36 ± 27.67	192.22 ± 32.35	0.014*
FBG (mmol/L)	5.20 ± 0.65	5.32 ± 0.77	0.604
CREA (mmol/L)	68.60 ± 26.26	72.48 ± 16.42	0.502
URIC (mmol/L)	405.27 ± 50.35	458.42 ± 99.01	0.085
TG (mmol/L)	1.56 ± 0.67	1.84 ± 1.01	0.379
TC (mmol/L)	4.02 ± 1.03	4.77 ± 1.28	0.067
HDL-C (mmol/L)	1.11 ± 0.19	1.10 ± 0.24	0.838
LDL-C (mmol/L)	2.72 ± 0.82	2.67 ± 0.61	0.209
eGFR (ml/min/1.73 m^2^)	102.57 ± 16.34	105.53 ± 14.67	0.579
SBP (mmHg)	139.82 ± 23.76	132.64 ± 16.37	0.352
DBP (mmHg)	96.00 ± 15.90	90.38 ± 11.80	0.281
FMD (%)	7.17 ± 0.81	4.68 ± 0.91	< 0.001^#^

Values are expressed as mean ± SD or number of participants (%)

*FMD* flow-mediated dilatation, *BMI* body mass index, *HGB* hemoglobin, *FBG* fasting blood glucose, *CREA* creatinine, *URIC* uric acid, *TG* triglycerides, *TC* total cholesterol, *HDL-C* high-density lipoprotein-cholesterol, *LDL-C* low-density lipoprotein-cholesterol, *eGFR* estimated glomerular filtration rate, *SBP* systolic blood pressure, *DBP* diastolic blood pressure

* represents *P* < 0.05

^#^ represents *P* < 0.01

### Risk factors for impaired FMD in the participants

Univariate and multivariate regression analyses were performed to identify potential risk factors for impaired FMD in hypertensive patients. According to the results of univariate analysis, only HGB levels were significantly associated with impaired FMD (OR = 1.030, 95% CI 1.004–1.057). After adjusting for confounding factors in the multivariate analysis, this association remained statistically significant (OR = 1.034, 95% CI 1.001–1.069, Table 4).

**Table 4:** Logistic regression analysis of impaired FMD and risk factors in the whole population

Risk factors	Model 1	Model 2
Age (years)	1.010 (0.903–1.129)	1.035 (0.896–1.196)
Male	2.630 (0.460–15.044)	1.889 (0.142–15.160)
Smoking	1.797 (0.442–7.309)	0.621 (0.104–3.720)
Drinking	1.367 (0.384–4.859)	1.869 (0.361–9.678)
BMI (kg/m^2^)	1.025 (0.870–1.208)	1.016 (0.825–1.251)
HGB (g/L)	1.030 (1.004–1.057)*	1.034 (1.001–1.069)*
FBG (mmol/L)	1.252 (0.539–2.908)	1.408 (0.404–4.910)
CREA (mmol/L)	1.012 (0.978–1.048)	0.982 (0.931–1.037)
URIC (mmol/L)	1.007 (0.999–1.015)	1.009 (0.999–1.019)
LDL-C (mmol/L)	0.872 (0.318–2.390)	0.738 (0.216–2.526)
SBP (mmHg)	0.979 (0.947–1.012)	0.988 (0.949–1.029)

Model 1: Univariate logistic regression analysis

Model 2: Multivariate logistic regression analysis: age, gender (male or female), smoking status (yes or no), drinking status (yes or no), BMI, HGB, FBG, CREA, URIC, LDL-C, and SBP

*BMI* body mass index, *HGB* hemoglobin, *FBG* fasting blood glucose, *CREA* creatinine, *URIC* uric acid, *LDL-C* low-density lipoprotein-cholesterol, *SBP* systolic blood pressure

* represents *P* < 0.05

## Discussion

To the best of our knowledge, this is the first study focusing on hypertensive individuals with concomitant HAPC, to investigate the effects of HAPC on HMODs in hypertensive patients. The main findings of our study can be summarized as follows: (1) After matching for age and gender, hypertensive patients with HAPC exhibited a significantly larger LA diameter and a higher proportion of individuals with impaired FMD compared to those without HAPC. This suggests that HAPC may exacerbate the severity of HMODs in hypertensive patients; (2) After adjusting for other confounding factors, peripheral HGB concentration remained significantly associated with LA diameter and impaired FMD, indicating that HGB is an independent risk factor for certain HMODs parameters in hypertensive patients with HAPC.

The assessment of HMODs is crucial for risk stratification and the management of hypertensive patients. LA abnormity represents a common form of HMOD and has been proved to play a significant role in predicting cardiovascular outcomes in a wide range of diseases [16]. In this study, we compared several echocardiographic parameters between hypertensive patients with and without HAPC. Our findings indicated that only the LA size was significantly larger in hypertensive patients with HAPC than those without HAPC. However, the LVMI and RWT, which reflect the geometric changes in the left ventricle (LV), exhibited no significant difference between the two groups. This observation aligns with previous studies on patients with polycythemia vera [17]. The disparity in LA and LV changes observed may be attributed to the unique physiological characteristics of the LA. The LA plays a central role in the entire circulatory system, receiving blood from the pulmonary veins, storing it, and then transferring it into the left ventricle. Throughout the cardiac cycle, it serves as a pump, reservoir, and conduit [18]. The size of LA is primarily influenced by two factors: pressure and volume. The LA is particularly sensitive to peripheral BP fluctuations, and LA enlargement is often one of the earliest cardiac structural changes observed in hypertensive patients [19]. In patients with HAPC, elevated HGB levels and increased blood viscosity contribute to heightened systemic circulation pressure [20, 21]. As a result, the LA may undergo more pronounced morphological changes than the LV. To be mentioned, after adjusting for SBP and other confounding factors, we found that HGB remained significantly associated with the LA size in our study. It indicates that except for increased peripheral resistance and volume overload, other mechanisms may be influencing the cardiac structure in patients with HAPC. Potential contributors include enhanced systemic inflammation and oxidative stress [22], autonomic dysregulation [6], and altered hemorheology caused by increased blood viscosity [23]. Further studies are warranted to confirm the specific underlying mechanisms.

In addition, we also evaluated the vascular endothelial function in the study population. It has been well-established that endothelium plays a key role in regulating vascular tone by releasing some vasoactive substances such as nitric oxide (NO) and prostaglandins. FMD values reflect the capacity of endothelium to produce NO and induce vasodilation in response to increased blood flow stimulus [24]. Impaired brachial FMD has now been widely recognized as an early marker of vascular disease and a potential indicator of target organ damage in hypertensive individuals [25]. Previous studies have demonstrated that FMD holds predictive value for cardiovascular events and mortality, regardless of an individual’s cardiovascular disease history [26, 27]. In high-altitude populations, Bailey DM et al. reported that individuals with CMS exhibited significantly lower FMD than healthy controls, indicating that CMS can also induce vascular dysfunction [28]. However, few studies have investigated the vascular health profiles of hypertensive individuals with concurrent CMS. In this study, after case–control matching, we found that hypertensive patients with HAPC showed significantly lower FMD values and a higher proportion of impaired FMD (< 6.5%) compared to those without HAPC. Moreover, according to the results of multivariate regression analysis, HGB was significantly correlated with impaired FMD when other confounding factors were adjusted. Our findings suggest that HAPC may exacerbate vascular dysfunction in hypertensive patients. Several mechanisms may be involved in this process.

First, altered sheer stress resulting from increased blood viscosity may contribute to the reduction in FMD. Tremblay JC et al. [29] conducted a study involving 42 male Andeans permanently residing in the Pasco region, including 23 individuals with HAPC. Their findings revealed that patients with HAPC exhibited reduced FMD and elevated blood viscosity compared to those non-HAPC individuals. Additionally, they observed significant associations between HGB levels, blood viscosity, and FMD in the population. Moreover, following isovolemic hemodilution, both HGB levels and blood viscosity decreased, accompanied by an improvement in FMD. These findings suggest a link between HGB/blood viscosity and FMD. Second, heightened adrenergic signaling may also be involved. Tymko et al. [30] reported that, when chronically exposed to high-altitude environment, lowlanders showed significantly reduced endothelial-dependent dilation (EDD). However, this impaired EDD was fully restored through adrenergic blockade. Notably, in highlanders with HAPC, adrenergic blockade could also partially alleviate the reduction in EDD, indicating that the upregulated adrenergic vasoconstrictor signaling plays a substantial role in hypoxia-induced endothelial dysfunction, which cannot be overlooked. Additionally, increased oxidative-nitrosative stress (ONS) has also been observed in patients with CMS. A study conducted in Bolivia reported that elevated ONS was associated with reduced FMD and increased AIx-75 [28], suggesting that this heightened systemic ONS response in CMS patients may partially contribute to the underlying mechanism of vascular dysfunction. Overall, previous findings highlighted the multifactorial nature of endothelial dysfunction in patients with HAPC, involving blood viscosity, adrenergic signaling, and oxidative-nitrosative stress. Further research is needed to fully elucidate these mechanisms.

### Limitations

Our study also has several limitations. First, fluctuations in BP can occur when patients descend from high altitudes to sea level. However, in this study, all BP measurements were taken at sea level, with no data on BP levels at high altitudes. It is not conducive to the evaluation of long-term BP load in patients at high altitudes. Second, erythropoietin levels were not assessed in the enrolled patients. Additionally, we assessed left atrial size using only left atrial end-diastolic diameter, without incorporating measurements of LA area or LA volume. Finally, while we observed that hypertensive patients with concomitant HAPC exhibited more pronounced HMODs, we did not explore the underlying mechanisms. Further research with a specific design is needed to elucidate the potential mechanisms.

## Conclusion

When matched for age and gender, hypertensive patients with HAPC showed significantly larger LA diameter and a higher proportion of individuals with impaired FMD compared to those without HAPC. Additionally, HGB seemed to be an independent risk factor for both increased LA diameter and impaired FMD in hypertensive patients with HAPC.

## Data Availability

The data that support the findings of this study are available on request from the corresponding author.

## Abbreviations

CMS: Chronic mountain sickness
HTN: Hypertension
HAPC: High-altitude polycythemia
ASCVDs: Atherosclerotic cardiovascular diseases
HMODs: Hypertension-mediated organ damages
FMD: Flow-mediated dilation
LA: Left atrium
LV: Left ventricle
IVS: Interventricular septal wall
LVPW: Left ventricular posterior wall
AAO: Ascending aorta
RWT: Relative wall thickness
LVM: Left ventricular mass
BSA: Body surface area
LVMI: Left ventricular mass index
LVH: Left ventricular hypertrophy
*E*: Early diastolic inflow velocity
*A*: Atrial systolic inflow velocity
*e*′: Early diastolic mitral annular velocity
IMT: Intima-media thickness
BP: Blood pressure
OBP: Office blood pressure
SBP: Systolic blood pressure
DBP: Diastolic blood pressure
BMI: Body mass index
HGB: Hemoglobin
FBG: Fasting blood glucose
CREA: Creatinine
UA: Uric acid
TC: Total cholesterol
TG: Triglycerides
HDL-C: High-density lipoprotein cholesterol
LDL-C: Low-density lipoprotein cholesterol
eGFR: Estimated glomerular filtration rate
EDD: Endothelial-dependent dilation
NO: Nitric oxide
ONS: Oxidative-nitrosative stress

## References

[1] Mallet RT, Burtscher J, Richalet JP, Millet GP, Burtscher M (2021) Impact of High Altitude on Cardiovascular Health: Current Perspectives. Vasc Health Risk manag 17:317–335. 10.2147/vhrm.S294121 34135590 10.2147/VHRM.S294121 PMC8197622

[2] Storz JF, Scott GR (2019) Life Ascending: Mechanism and Process in Physiological Adaptation to High-Altitude Hypoxia. Annu Rev Ecol Evol Syst 50:503–526. 10.1146/annurev-ecolsys-110218-025014 33033467 10.1146/annurev-ecolsys-110218-025014 PMC7540626

[3] León-Velarde F, Maggiorini M, Reeves JT, Aldashev A, Asmus I, Bernardi L, Ge RL, Hackett P, Kobayashi T, Moore LG et al (2005) Consensus statement on chronic and subacute high altitude diseases. High Alt Med Biol 6:147–157. 10.1089/ham.2005.6.147 16060849 10.1089/ham.2005.6.147

[4] Zhao X, Li S, Ba S, He F, Li N, Ke L, Li X, Lam C, Yan LL, Zhou Y et al (2012) Prevalence, awareness, treatment, and control of hypertension among herdsmen living at 4300 m in Tibet. Am J Hypertens 25:583–589. 10.1038/ajh.2012.9 22357415 10.1038/ajh.2012.9

[5] Vasan RS, Song RJ, Xanthakis V, Beiser A, DeCarli C, Mitchell GF, Seshadri S (2022) Hypertension-mediated organ damage: prevalence, correlates, and prognosis in the community. Hypertension 79:505–515. 10.1161/hypertensionaha.121.18502 35138872 10.1161/HYPERTENSIONAHA.121.18502 PMC8849561

[6] Richalet JP, Hermand E, Lhuissier FJ (2024) Cardiovascular physiology and pathophysiology at high altitude. Nat Rev Cardiol 21:75–88. 10.1038/s41569-023-00924-9 37783743 10.1038/s41569-023-00924-9

[7] Corante N, Anza-Ramírez C, Figueroa-Mujíca R, Macarlupú JL, Vizcardo-Galindo G, Bilo G, Parati G, Gamboa JL, León-Velarde F, Villafuerte FC (2018) Excessive Erythrocytosis and Cardiovascular Risk in Andean Highlanders. High Alt Med Biol 19:221–231. 10.1089/ham.2017.0123 29782186 10.1089/ham.2017.0123 PMC6157350

[8] Chen C, Lu FC (2004) The guidelines for prevention and control of overweight and obesity in Chinese adults. Biomed Environ Sci 17(Suppl):1–36 15807475

[9] Williams B, Mancia G, Spiering W, Agabiti Rosei E, Azizi M, Burnier M, Clement DL, Coca A, de Simone G, Dominiczak A et al (2018) 2018 ESC/ESH Guidelines for the management of arterial hypertension. Eur Heart J 39:3021–3104. 10.1093/eurheartj/ehy339 30165516 10.1093/eurheartj/ehy339

[10] Inker LA, Eneanya ND, Coresh J, Tighiouart H, Wang D, Sang Y, Crews DC, Doria A, Estrella MM, Froissart M et al (2021) New Creatinine- and Cystatin C-Based Equations to Estimate GFR without Race. N Engl J Med 385:1737–1749. 10.1056/NEJMoa2102953 34554658 10.1056/NEJMoa2102953 PMC8822996

[11] Sun Y, Sun J, Zhang P, Zhong F, Cai J, Ma A. Association of dietary fiber intake with hyperuricemia in U.S. adults. Food & function 2019;10:4932–4940. 10.1039/c8fo01917g 10.1039/c8fo01917g 31342970

[12] Mitchell C, Rahko PS, Blauwet LA, Canaday B, Finstuen JA, Foster MC, Horton K, Ogunyankin KO, Palma RA, Velazquez EJ (2019) Guidelines for Performing a Comprehensive Transthoracic Echocardiographic Examination in Adults: Recommendations from the American Society of Echocardiography. J Am Soc Echocardiogr 32:1–64. 10.1016/j.echo.2018.06.004 30282592 10.1016/j.echo.2018.06.004

[13] Lang RM, Badano LP, Mor-Avi V, Afilalo J, Armstrong A, Ernande L, Flachskampf FA, Foster E, Goldstein SA, Kuznetsova T et al (2015) Recommendations for cardiac chamber quantification by echocardiography in adults: an update from the American Society of Echocardiography and the European Association of Cardiovascular Imaging. J Am Soc Echocardiogr 28:1-39.e14. 10.1016/j.echo.2014.10.003 25559473 10.1016/j.echo.2014.10.003

[14] Thijssen DHJ, Bruno RM, van Mil A, Holder SM, Faita F, Greyling A, Zock PL, Taddei S, Deanfield JE, Luscher T et al (2019) Expert consensus and evidence-based recommendations for the assessment of flow-mediated dilation in humans. Eur Heart J 40:2534–2547. 10.1093/eurheartj/ehz350 31211361 10.1093/eurheartj/ehz350

[15] Heiss C, Rodriguez-Mateos A, Bapir M, Skene SS, Sies H, Kelm M (2023) Flow-mediated dilation reference values for evaluation of endothelial function and cardiovascular health. Cardiovasc Res 119:283–293. 10.1093/cvr/cvac095 35709326 10.1093/cvr/cvac095

[16] Hoit BD (2014) Left atrial size and function: role in prognosis. J Am Coll Cardiol 63:493–505. 10.1016/j.jacc.2013.10.055 24291276 10.1016/j.jacc.2013.10.055

[17] Jóźwik-Plebanek K, Dobrowolski P, Lewandowski J, Narkiewicz K, Sikorska A, Siński M, Eisenhofer G, Schmieder RE, Januszewicz M, Windyga J et al (2020) Blood pressure profile, sympathetic nervous system activity, and subclinical target organ damage in patients with polycythemia vera. Pol Arch Intern Med 130:607–614. 10.20452/pamw.15473 32621668 10.20452/pamw.15473

[18] Pagel PS, Kehl F, Gare M, Hettrick DA, Kersten JR, Warltier DC (2003) Mechanical function of the left atrium: new insights based on analysis of pressure-volume relations and Doppler echocardiography. Anesthesiology 98:975–994. 10.1097/00000542-200304000-00027 12657862 10.1097/00000542-200304000-00027

[19] Salas Pacheco JL, Sánchez OL (2019) Independent parameters of left atrium function in hypertensive heart disease. Echocardiography 36:2195–2201. 10.1111/echo.14542 31755581 10.1111/echo.14542

[20] Fowkes FG, Lowe GD, Rumley A, Lennie SE, Smith FB, Donnan PT (1993) The relationship between blood viscosity and blood pressure in a random sample of the population aged 55–74 years. Eur Heart J 14:597–601. 10.1093/eurheartj/14.5.597 8508852 10.1093/eurheartj/14.5.597

[21] Cinar Y, Demir G, Paç M, Cinar AB (1999) Effect of hematocrit on blood pressure via hyperviscosity. Am J Hypertens 12:739–743. 10.1016/s0895-7061(99)00011-4 10411372 10.1016/s0895-7061(99)00011-4

[22] Yi H, Yu Q, Zeng D, Shen Z, Li J, Zhu L, Zhang X, Xu Q, Song H, Kong P (2021) Serum Inflammatory Factor Profiles in the Pathogenesis of High-Altitude Polycythemia and Mechanisms of Acclimation to High Altitudes. Mediators Inflamm 2021:8844438. 10.1155/2021/8844438 34483727 10.1155/2021/8844438 PMC8413029

[23] Cowan AQ, Cho DJ, Rosenson RS (2012) Importance of blood rheology in the pathophysiology of atherothrombosis. Cardiovasc Drugs Ther 26:339–348. 10.1007/s10557-012-6402-4 22821616 10.1007/s10557-012-6402-4

[24] Modena MG, Bonetti L, Coppi F, Bursi F, Rossi R (2002) Prognostic role of reversible endothelial dysfunction in hypertensive postmenopausal women. J Am Coll Cardiol 40:505–510. 10.1016/s0735-1097(02)01976-9 12142118 10.1016/s0735-1097(02)01976-9

[25] Gkaliagkousi E, Gavriilaki E, Triantafyllou A, Douma S (2015) Clinical Significance of Endothelial Dysfunction in Essential Hypertension. Curr Hypertens Rep 17:85. 10.1007/s11906-015-0596-3 26371063 10.1007/s11906-015-0596-3

[26] Yeboah J, Crouse JR, Hsu FC, Burke GL, Herrington DM (2007) Brachial flow-mediated dilation predicts incident cardiovascular events in older adults: the Cardiovascular Health Study. Circulation 115:2390–2397. 10.1161/circulationaha.106.678276 17452608 10.1161/CIRCULATIONAHA.106.678276

[27] Yeboah J, Folsom AR, Burke GL, Johnson C, Polak JF, Post W, Lima JA, Crouse JR, Herrington DM (2009) Predictive value of brachial flow-mediated dilation for incident cardiovascular events in a population-based study: the multi-ethnic study of atherosclerosis. Circulation 120:502–509. 10.1161/circulationaha.109.864801 19635967 10.1161/CIRCULATIONAHA.109.864801 PMC2740975

[28] Bailey DM, Rimoldi SF, Rexhaj E, Pratali L, Salinas Salmòn C, Villena M, McEneny J, Young IS, Nicod P, Allemann Y et al (2013) Oxidative-nitrosative stress and systemic vascular function in highlanders with and without exaggerated hypoxemia. Chest 143:444–451. 10.1378/chest.12-0728 22922469 10.1378/chest.12-0728

[29] Tremblay JC, Hoiland RL, Howe CA, Coombs GB, Vizcardo-Galindo GA, Figueroa-Mujíca RJ, Bermudez D, Gibbons TD, Stacey BS, Bailey DM et al (2019) Global REACH 2018: High Blood Viscosity and Hemoglobin Concentration Contribute to Reduced Flow-Mediated Dilation in High-Altitude Excessive Erythrocytosis. Hypertension 73:1327–1335. 10.1161/hypertensionaha.119.12780 31006327 10.1161/HYPERTENSIONAHA.119.12780

[30] Tymko MM, Lawley JS, Ainslie PN, Hansen AB, Hofstaetter F, Rainer S, Amin S, Moralez G, Gasho C, Vizcardo-Galindo G et al (2020) Global Reach 2018 Heightened α-Adrenergic Signaling Impairs Endothelial Function During Chronic Exposure to Hypobaric Hypoxia. Circ Res 127:e1–e13. 10.1161/circresaha.119.316053 32268833 10.1161/CIRCRESAHA.119.316053 PMC7483295

